# Square Wave Voltammetric Determination of Diclofenac in Pharmaceutical Preparations and Human Serum

**Published:** 2015

**Authors:** Ulvihan Ciltas, Bilal Yilmaz, Selcuk Kaban, Bilge Kaan Akcay, Gulsah Nazik

**Affiliations:** *Department of Analytical Chemistry, School of Pharmacy, Ataturk University, Erzurum, Turkey.*

**Keywords:** Diclofenac, Cyclic voltammetry, Square wave voltammetry, Pharmaceutical tablet, Serum

## Abstract

In this study, a simple and reliable square wave voltammetric (SWV) method was developed and validated for determination of diclofenac in pharmaceutical preparations and human serum. The proposed method was based on electrooxidation of diclofenac at platinum electrode in 0.1 M TBAClO_4_/acetonitrile solution. The well-defined two oxidation peaks were observed at 0.87 and 1.27 V, respectively. Calibration curves that were obtained by using current values measured for second peak were linear over the concentration range of 1.5-17.5 μg mL^-1 ^and 2-20 μg mL^-1 ^ in supporting electrolyte and serum, respectively. Precision and accuracy were also checked in all media. Intra- and inter-day precision values for diclofenac were less than 3.64, and accuracy (relative error) was better than 2.49%. Developed method in this study is accurate, precise and can be easily applied to Diclomec, Dicloflam and Voltaren tablets as pharmaceutical preparation. Also, the proposed technique was successfully applied to spiked human serum samples. No electroactive interferences from the endogenous substances were found in human serum.

## Introduction

**Figure 1:**
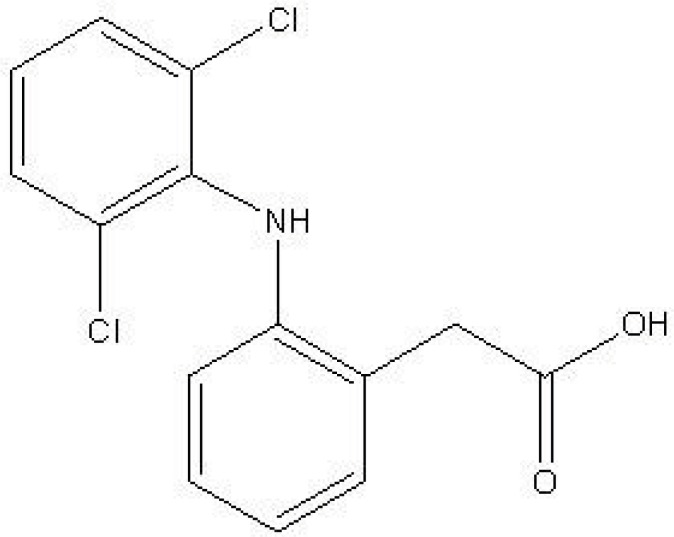
Chemical structure of diclofenac.

Diclofenac (Figure 1) is a nonsteroidal anti-inflammatory drug (NSAID) that is widely prescribed for the treatment of rheumatoid arthritis, osteoarthritis, musculoskeletal injuries and post surgery analgesia in human and veterinary medicine. Patients are frequently given special formulations of diclofenac or a co-treatment agent as a therapeutic strategy to attenuate the gastrointestinal tract complications that limit the use of diclofenac and other NSAIDs (1-3). Many patients prescribed diclofenac for arthritis also take additional drugs for other chronic health problems such as hypertension (4,5).

Several methods have been reported for determination of diclofenac including gas chromatography-mass spectrometry (GC-MS) (6-8), high-performance liquid chromatography (HPLC) (9-23) and LC-MS-MS (24) in human plasma and other biological fluids.

The reported methods were influenced by interference of endogenous substances and potential loss of drugs in the re-extraction procedure and involving lengthy, tedious and time-onsuming plasma sample preparation and extraction processes and requiring a sophisticated and expensive instrumentation. 

The development of a new method capable of determining drug amount in pharmaceutical dosage forms is important. Electroanalytical techniques have been used for the determination of a wide range of drug compounds with the advantages that there are, in most, instances no need for derivatization and that these techniques are less sensitive to matrix effects than other analytical techniques. Additionally, application of electrochemistry includes the determination of electrode mechanism. Redox properties of drugs can give insights into their metabolic fate or their *in-vivo* redox processes or pharmacological activity. Despite the analytical importance of the electrochemical behavior and oxidation mechanism of diclofenac, no report has been published on the voltammetric study of the electrochemical oxidation of diclofenac in nonaqueous media. It is well known that the experimental and instrumental parameters directly affect the electrochemical process and voltammetric response of drugs. Consequently, it would be interest to investigate the oxidation process of diclofenac in aprotic media. Therefore, the goal of this work was the development of new SWV method for the direct determination of diclofenac in pharmaceutical preparations and spiked human serum samples without any time-consuming extraction or evaporation steps prior to drug assay. This paper describes fully validated, simple, rapid, selective and sensitive procedures for the determination of diclofenac employing SWV at the platinum disc electrode. Besides, the proposed method might be alternatives to the chromatographic methods in therapeutic drug monitoring.

## Experimental

*Chemical* *and reagents*

Diclofenac (sodium salt) was obtained from Sigma (St. Louis, MO, USA). Acetonitrile (Fluka for HPLC analysis) was purified by drying with calcium hyride, followed by distillation from phosphorus pentoxide. After purification in order to eliminate its water content as much as possible, it was kept over molecular sieves (3Å, Merck). Tetrabutylammonium perchlorate (TBAClO_4_) were purchased from Fluka and used as received without further purification. Diclomec, Dicloflam and Voltaren tablets were obtained from pharmacy (Erzurum, Turkey). Distilled water was prepared as required by using aquaMAX™ ultra, Young instrument (Korea) ultrawater purification system. Human serum was obtained from Yakutiye blood bank, Erzurum, Turkey.

*Electrochemical instrumentation*

Electrochemical experiments were performed on a Gamry Potentiostat Interface 1000 controlled with software PHE 200 and PV 220. All measurements were carried out in a single-compartment electrochemical cell with a standard three-electrode arrangement. A platinum disk with an area of 0.72 cm^2^ and a platinum wire were used as the working and the counter electrodes, respectively. The working electrode was successively polished with 1.0, 0.3 and 0.05 µm alumina slurries (Buehler) on microcloth pads (Buehler). After each polishing, the electrode was washed with water and sonicated for 10 min in acetonitrile. Then, it was immersed into a hot piranha solution (3:1, H_2_SO_4_, 30% H_2_O_2_) for 10 min, and rinsed copiously with water. Caution: Piranha is a vigorous oxidant and should be used with extreme caution! All potentials were reported versus Ag/AgCl/KCl (3.0 M) reference electrode (BAS Model MF-2078) at room temperature. The electrolyte solutions were degassed with purified nitrogen for 10 min before each experiment and bubbled with nitrogen during the experiment. Operating conditions for SWV were pulse amplitude 25 mV, frequency 15 Hz, potential step 4 mV.

Preparation of the Standard and Quality Control Solutions 

The stock standard solution of diclofenac was prepared in 0.1 M TBAClO_4_/acetonitrile to a concentration of 100 g mL^-1^ and stored at 4  C. Working standard solutions were prepared from the stock solution. A calibration graph was constructed in the range of 1.5, 2.5, 5, 7.5, 10, 12.5, 15 and 17.5 g mL^-1 ^for diclofenac (n=6). For quality control (QC) samples containing concentration 4, 8 and 16 g mL^-1 ^of diclofenac, the stock solution was diluted with 0.1 M TBAClO_4_/acetonitrile.

*Procedure for pharmaceutical preparations*

A total of 10 tablets of diclofenac (Diclomec, Dicloflam and Voltaren) accurately weighed and powdered. An amount of this powder corresponding to one tablet diclofenac content was weighed and accurately transferred into 100 mL calibrated flask and 50 mL of 0.1 M TBAClO_4_/acetonitrile was added and then the flask was sonicated to 10 min at room tempature. The flask was filled to volume with 0.1 M TBAClO_4_/acetonitrile. The resulting solutions in both the cases were filtered through Whatman filter paper no 42 and suitably diluted to get final concentration within the limits of linearity for the respective proposed method. The drug content of diclofenac tablets was calculated from the current potential curve. 

*Recovery studies*

To study the accuracy and reproducibility of the proposed technique, recovery experiments were carried out using the standard addition method. In order to know whether the excipients show any interference with the analysis, known amounts of pure diclofenac were added to the pre-analyzed tablet formulation and the mixtures were analyzed by the proposed method. After five repeated experiments, the recovery results were calculated using the calibration equation.

*Analysis of spiked serum samples*

Serum samples, obtained from healthy individuals (after obtaining their written consent) were stored frozen until assay. After gentle, thawing, an aliquot volume of sample was fortified with diclofenac dissolved in bi-distilled water to achieve final concentration of 100 μg mL^-1 ^and treated with 0.7 mL of acetonitrile as serum denaturating and precipitating agent, then the volume was completed to 2 mL with the same serum sample. The tubes were vortexed for 10 min and then centrifuged for 5 min at 5000 × *g *for removing of protein residues. The supernatant was taken carefully. 

The concentration of diclofenac was varied in the range of 2 to 20 μg mL^-1 ^in human serum samples. These solutions were analyzed in the voltammetric cell containing 0.1 M TBAClO_4_/acetonitrile. The amount of diclofenac in spiked human serum samples for the recovery studies was calculated from the related calibration equation.

## Results and Discussion

*Voltammetric behavior of diclofenac*

**Figure 2:**
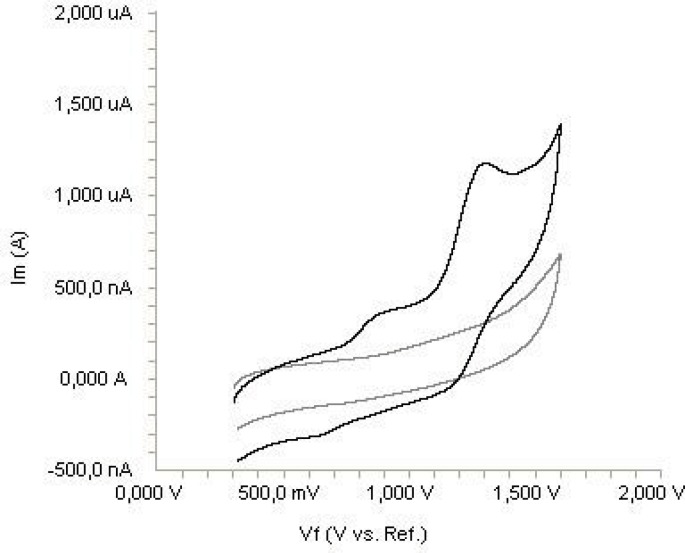
Cyclic voltammogram for the oxidation of 20 μg mL^-1^ diclofenac in acetonitrile containing 0.1 M TBAClO_4_ at Pt disk electrode, scan rate: 0.2 V s^-1^.

The electrochemical behavior of diclofenac was investigated at the Pt disc electrode in anhydrous acetonitrile solution containing 0.1 M TBAClO_4 _as the supporting electrolyte by using cyclic voltammetry (CV). Figure 2 shows a typical cyclic voltammogram of 20 μg mL^-1 ^diclofenac recorded under these conditions for the scan rate of 0.2 V s^-1^. In the anodic sweep, two oxidation peaks are seen at about potentials of 0.87 and 1.27 V, respectively. 

**Figure 3:**
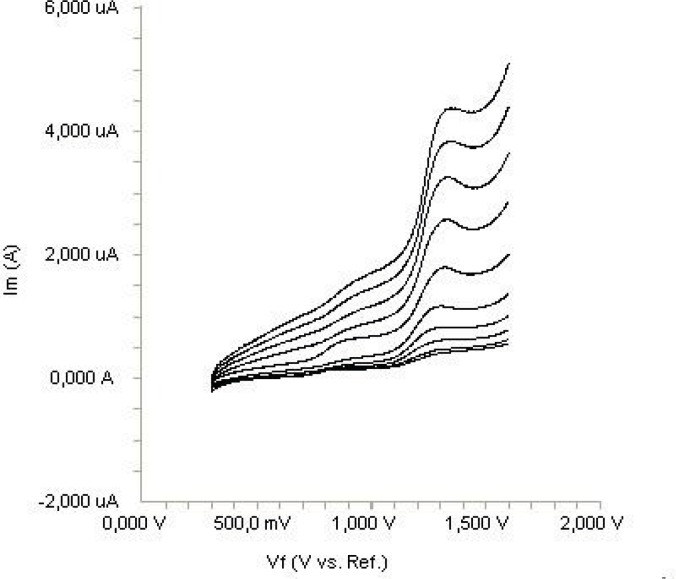
Linear sweep voltammograms for the oxidation of 20 μg mL^-1^ diclofenac in acetonitrile containing 0.1 M TBAClO_4 _as a function of scan rate.

**Figure 4:**
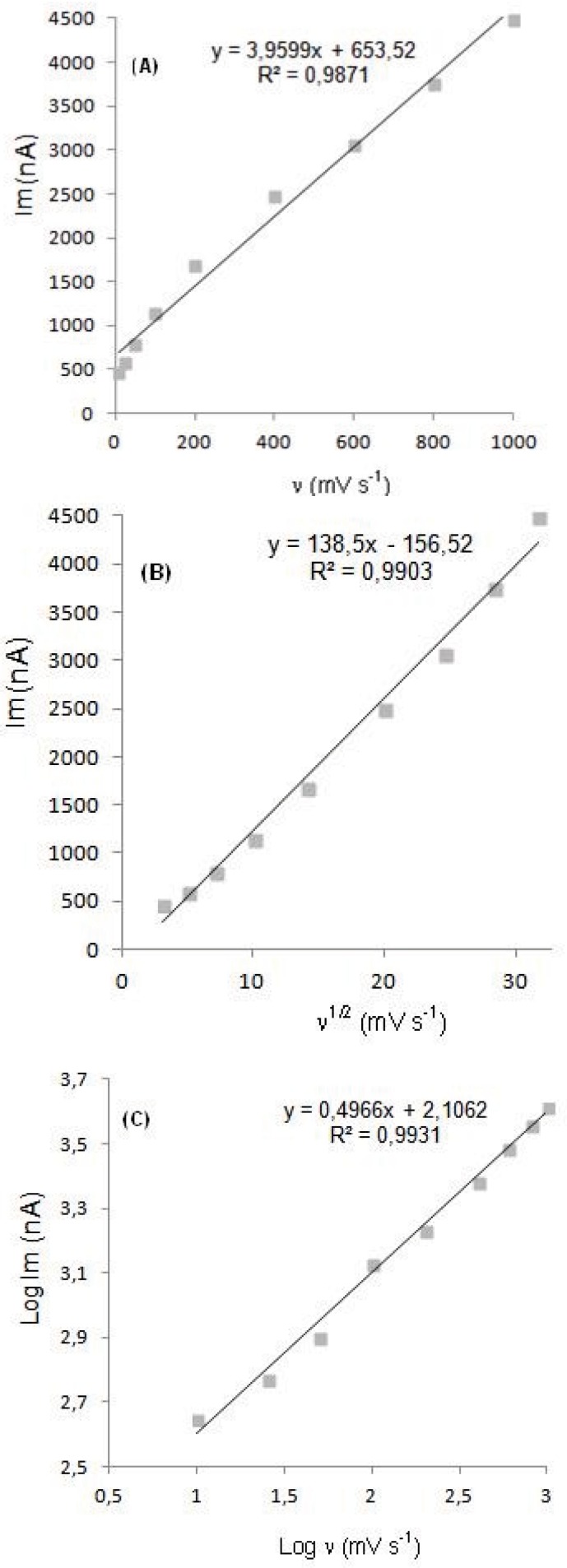
Dependence of peak current on the scan rate (20 μg mL^-1^).

In order to gain a deeper insight into the voltammetric waves, the effect of scan rate on the anodic peak currents (*İ*_m_) and peak potentials (E_p_) was studied in the range of 0.01-1 V s^-1^ of the potential scan rates in acetonitrile solution containing 20 μg mL^-1^ concentration of diclofenac (Figure 3). The representative linear sweep voltammograms obtained at Pt electrode for 20 μg mL^-1 ^diclofenac as a function of the scan rate are presented in Figure 4. Scan rate dependency experiments show that the peak currents for peak vary linearly with the scan rate (ν) (Figures 4a,b), which points out the adsorption-controlled process. However, the plots of logarithm of peak currents versus logarithm of scan rates for 20 μg mL^-1^ concentration of diclofenac display straight lines with 0.497 slope (Figure 4c), which are close to theoretical value of 0.5 expected for an ideal diffusion-controlled electrode process (25). Log I_m_-log ν curve is more eligible for this aim, therefore, a diffusional process for peak should be considered. These results suggest that the redox species are diffusing freely from solution and not precipitating onto the electrode surface. The reason for this behavior may be due to the solubility of the intermediate species in acetonitrile or poor adherence of products on the electrode surface.

*Analytical applications and validation of the proposed method*

In order to develop a voltammetric procedure for determination of the drug, we selected the SWV technique, since the peaks were sharper and better defined at lower concentration of diclofenac than those obtained by cyclic and linear sweep voltammetry with a lower background current, resulting in improved resolution. SWV is effective and rapid electroanalytical techniques with well-established advantages, including good discrimination against background currents and low detection limits (26,27). 

**Figure 5:**
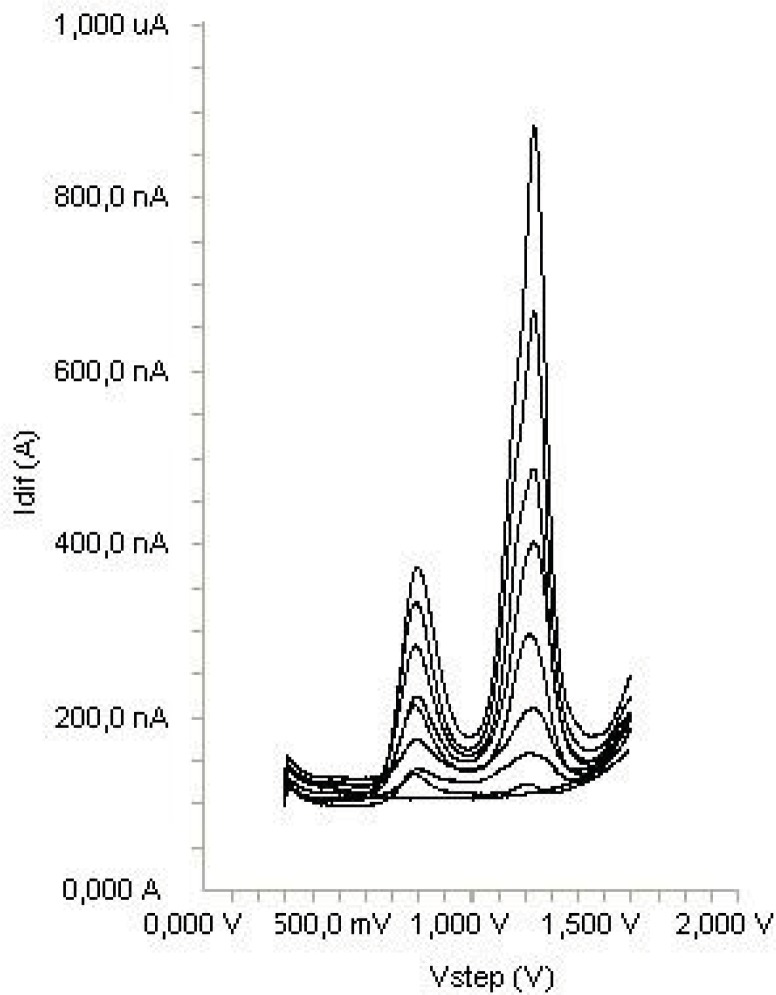
SWV voltammograms obtained for the determination in supporting electrolyte (blank, 1.5, 2.5, 5, 7.5, 10, 12.5, 15 and 17.5  g mL^-1^).

Calibration graphs from the standard solution of diclofenac according to the procedures described above was constructed by using SWV. A linear relation in the concentration range between 1.5-17.5 μg mL^-1 ^was found, indicating that the response was diffusion controlled in this range (Figure 5). Above this concentration (17.5 μg mL^-1^) a loss of linearity was probably due to the adsorption of diclofenac on the electrode surface. The characteristics of the calibration plots are summarized in Table 1. The limit of detection (LOD) and the limit of quantification (LOQ) were calculated on the peak current using the following equations:

LOD = 3 s*/*m; LOQ = 10 s*/*m

where s is the standard deviation of the peak currents (three runs) and m is the slope of the calibration curve. The LOD and LOQ values were also shown is Table 1. Repeating five experiments on 15 μg mL^-1 ^diclofenac for SWV technique tested the repeatability and reproducibility of peak potential and peak currents. The results were shown also in Table 1. Repetition of sample analysis after 72 h period did not show any significant change in results of analyses.

**Table 1:** Regression data of the calibration lines for quantitative determination of diclofenac.

**Parameters**	**SWV**	
	**Supporting electrolyte**	**Serum**
Measured potential (V)	1.24	1.29
Linearity (μg mL^-1^)	1.5-17.5	2-20
Slope	7.8094	6.9425
Intercept	87.52	95.43
R	0.998	0.997
Sa	10.64	23.46
Sb	0.625	1.247
LOD (μg mL^-1^)	0.50	0.67
LOQ (μg mL^-1^)	1.50	2.00
Repeatability of peak current (RSD%)^a^	1.25	2.35
Repeatability of peak potential (RSD%)	1.34	1.94
Reproducibility of peak current (RSD%)	2.34	2.39
Reproducibility of peak potential (RSD%)	0.64	0.92

RSD: Relative standard derivation,

a Average of six replicate determinations, Sa: Standard deviation of intercept of regression line, Sb: Standard deviation of slope of regression line, R: Coefficient of correlation, LOD: Limit of detection, LOQ: Limit of quantification

*Determination of d* *iclofenac* *in tablets*

On the basis of above results, SWV method was applied to the direct determination of diclofenac in pharmaceutical preparations, using the related calibration straight lines without any sample extraction or filtration and after an adequate dilutions. The results show that the proposed method was successfully applied for the assay of diclofenac in its pharmaceutical dosage forms (Table 2). The accuracy of the method was determined by its recovery during spiked experiments. Recovery studies were carried out after the addition of known amounts of the pure drug to various pre-analyzed formulation of diclofenac. According to the results, excipients presented in tablet do not interfere with the analysis (Table 2). 

**Table 2:** Recovery of diclofenac in pharmaceutical preparations.

	**Diclomec**	**Dicloflam**	**Voltaren**
Labeled claim (mg)	100	50	75
Amount found (mg)^a^	99.8	50.4	75.6
RSD%	2.13	1.97	2.68
Bias%	-0.2	0.8	0.8
Added (mg)	10	10	10
Found (mg)	9.98	10.12	10.09
Recovery%	99.8	101.2	100.9
RSD% of recovery	2.45	1.98	2.04

a Each value is the mean of five experiments

There is no official method in any pharmacopoeias (*e.g*. USP, BP or EP) or literature method related to pharmaceutical dosage forms of diclofenac. To prove the absence of interferences by excipients, recovery studies were carried out. The results demonstrate the validity of the proposed method for the determination of diclofenac in tablets. These results reveal that both methods had adequate precision and accuracy and consequently can be applied to the determination of diclofenac in pharmaceuticals without any interference from the excipients.

*Determination of diclofenac in human serum samples*

**Figure 6:**
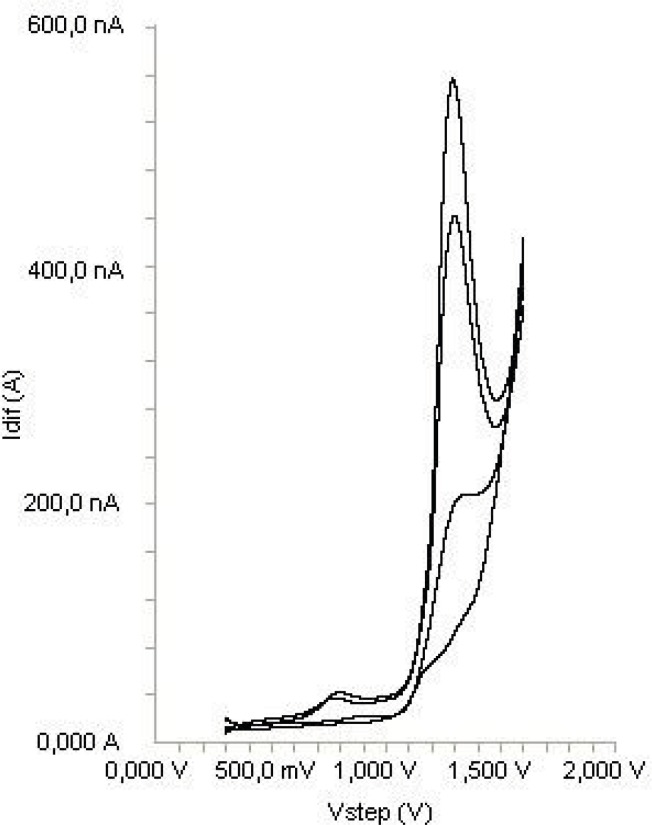
SWV voltammograms obtained for the determination in spiked serum (blank, 5, 10 and 15  g mL^-1^).

Acetonitrile and methanol were tried as a serum precipitating agents. Also, different amount of acetonitrile were tried. The best results were obtained using 0.7 mL acetonitrile. The measurements of diclofenac in serum samples were performed as described in Section 2. The applicability of the proposed method to the human serum, the calibration equations were obtained in spiked serum samples. Calibration equation parameters and necessary validation data were shown in Table 1. Obtained recovery results of spiked serum samples were given in Table 3. Analysis of drugs from serum samples usually requires extensive time-consuming sample preparation, use of expensive organic solvents and other chemicals. In this study, the serum proteins and endogenous substances in serum samples are precipitated by the addition of acetonitrile, which is centrifuged at 5000×*g*, and the supernatant was taken and diluted with the supporting electrolyte and directly analyzed. Typical SWV curve of diclofenac examined in serum samples are shown in Figure 6.

Using SWV technique, no sample pre-treatment was required, other than precipitation and dilution steps. The recovery results of diclofenac (Table 3) in serum samples were calculated from the related linear regression equations, which are given in Table 1. As it can be seen in Figure 6, no oxidation compounds and no extra noise peaks present in biological material peak occurred in the potential range where the analytical peak appeared. 

Stability of serum samples kept in refrigerator (+4 ^0^C) was tested by making five consecutive analyses of the sample over a period of approximately 5 h. There were no significant changes in the peak currents and potentials between the first and last measurements.

**Table 3:** Recovery of diclofenac in human serum

**Added ** **(μg mL** ^-1^ **)**	**Found** **(Mean ** **± ** ** SD** a **)**	**% Recovery**	**% RSD** b
2	1.94 ± 0.052	97.0	2.68
5	4.86 ± 0.218	97.2	4.49
7.5	7.42 ± 0.504	87.2	6.79
10	9.78 ± 0.718	98.9	7.34
12.5	12.43 ± 1.09	99.4	8.77
15	14.76 ± 1.28	98.4	8.67
17.5	18.14 ± 1.36	103.6	7.49
20	20.1 ± 1.51	100.5	7.51

SDa : Standard deviation of six replicate determinations, RSD: Relative standard deviation

b Average of six replicate determinations

## Conclusion

SWV technique has been developed for the determination of diclofenac in pharmaceutical preparations and human serum samples. The results obtained show that the above-described methods are useful not only for diclofenac determination in conventional electrolytes, but also in more complex matrices such as dosage forms and human serum samples. The principal advantages of SWV technique over the other techniques are that they may be applied directly to the analysis of pharmaceutical dosage forms and biological samples without the need for separation or complex sample preparation, since there was no interference from the excipients and endogenous substances. The method is rapid, requiring less than 5 min to run sample.

This paper is not intended to be a study of the pharmacodynamic properties of diclofenac, since only healthy volunteers were used for the sample collection and results may be of no significance. Its only show that the possibility of monitoring this drug makes the method useful for pharmacokinetic and pharmacodynamic purposes.
